# Influence of Prosthetic Emergence Profile on Peri-Implant Marginal Bone Stability: A Comprehensive Review

**DOI:** 10.3390/medicina61030517

**Published:** 2025-03-17

**Authors:** Rossana Izzetti, Chiara Cinquini, Marco Nisi, Michele Covelli, Fortunato Alfonsi, Antonio Barone

**Affiliations:** 1 Department of Surgical, Medical, Molecular Pathology and of the Critical Area, University of Pisa, 56126 Pisa, Italystudiodralfonsi@gmail.com (F.A.); 2 Dental Biomaterials Researh Unit (d-BRU), University of Liege, 4020 Liege, Belgium; 3 Department of Medicine and Surgery, LUM University, 70010 Bari, Italy

**Keywords:** dental implants, emergence profile, marginal bone, prosthesis, stability

## Abstract

The prosthetic emergence profile is a factor potentially affecting marginal bone level around dental implants. The aim of this review is to provide a comprehensive analysis of the influence of the prosthetic emergence profile on peri-implant marginal bone-level stability. The marginal bone level is an important parameter in implant dentistry, reflecting the stability of dental implants, and it is a critical indicator of long-term implant success. Minimizing marginal bone loss around dental implants is a key factor for maintaining implant function, supporting peri-implant soft tissues, and achieving predictable aesthetic outcomes. The scientific literature presents various examples of evidence on the influence of emergence angle and prosthetic designs on marginal bone loss. Several studies suggest that emergence angles exceeding 30° and convex prosthetic designs may increase the risk of peri-implantitis and bone resorption, while others find no significant correlation. Moreover, several studies demonstrated the positive influence of taper joint connections on marginal bone stability. Although the current literature remains diverse, it is essential to prioritize cleanability and minimize plaque accumulation for a successful implant-prosthetic restoration. Proper maintenance and a continuous follow-up to monitor marginal bone loss are beneficial for obtaining stable and optimal long-term results.

## 1. Introduction

The influence of the prosthetic emergence profile on the marginal bone level is a critical consideration in implant dentistry, as it can significantly affect the stability and health of peri-implant tissues. The emergence profile refers to the contour of the implant-prosthetic rehabilitation as it transitions from the implant platform to the prosthetic crown. This design impacts both the mechanical and biological aspects of peri-implant tissue health, influencing outcomes such as marginal bone stability, soft tissue adaptation, and the overall aesthetic integration of the restoration. An optimized emergence profile minimizes undue stress on the surrounding tissues, promotes soft tissue attachment, and reduces the risk of inflammatory responses [1,2,3]. Several studies demonstrated that gradual and well-designed transitions in the emergence profile improve tissue sealing and limit bacterial colonization, which are crucial for marginal bone preservation [4,5].

Conversely, abrupt transitions or poorly designed prosthetic contours may result in the overloading of peri-implant tissues, leading to increased plaque accumulation, inflammatory reactions, and subsequent bone resorption [6]. The concept of biologic width, defined as the soft tissue dimension necessary for the stable integration of peri-implant tissues, is also intrinsically linked to prosthetic emergence design. The disruption of this critical zone can trigger bone remodeling as tissues attempt to re-establish a protective barrier [7]. Moreover, recent advances in digital workflows and CAD/CAM technologies have facilitated the precise customization of emergence profiles, offering a promising approach to optimize marginal bone preservation and long-term clinical outcomes [8,9,10].

The growing body of evidence underscores the necessity of integrating biologically driven and mechanically sound designs into the prosthetic phase of implant treatment. By prioritizing these aspects, clinicians can ensure enhanced peri-implant health, reduced complications, and improved patient satisfaction.

## 2. Features of Different Prosthetic Emergence Profiles

**Figure 1:**
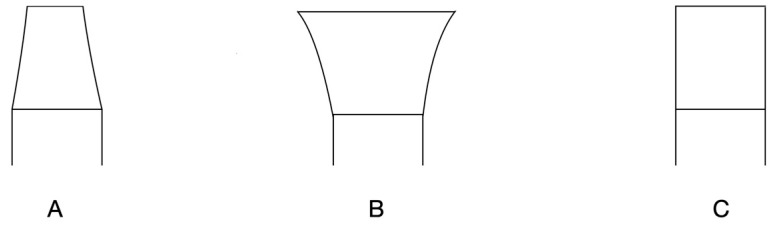
Schematic example of (**A**) concave, (**B**) convex, and (**C**) straight emergence profiles.

The features of prosthetic emergence profiles in implant dentistry are critical for achieving both functional success and aesthetic harmony, as they may influence the adaptation of peri-implant soft tissues and the preservation of marginal bone levels. Emergence profiles are classified primarily into concave, convex, and straight designs, each offering unique advantages and considerations. A schematic example of the various emergence profile designs is shown in Figure 1.

Concave emergence profiles taper inward from the implant platform to the prosthetic crown, creating space for soft tissue proliferation and improving vascularization, which enhances soft tissue thickness and stability. This design is particularly favorable in the anterior region, where aesthetics is a priority, as it facilitates a natural transition and minimizes marginal bone loss [4,11,12]. However, excessive concavity may compromise mechanical strength and make restoration maintenance more challenging [13].

Convex emergence profiles, characterized by their outward curvature, provide structural support to the soft tissues and may be suitable for areas requiring additional volume. However, this design can increase the risk of plaque accumulation and impair soft tissue adaptability if not properly tailored to the patient’s anatomy. Convex profiles are often used cautiously, as excessive pressure on the soft tissue may result in inflammation or bone remodeling [14,15]. Straight profiles, offering a neutral and linear transition between the implant platform and the prosthetic crown, are commonly employed in posterior regions where aesthetics are less critical but functional and hygienic considerations dominate. This design is easier to maintain and poses minimal impact on peri-implant tissues, making it a practical choice in many clinical scenarios [16].

The selection of the appropriate emergence profile depends on various factors, including the patient’s soft tissue biotype, implant position [17], prosthetic requirements, and aesthetic demands. Thin biotypes often benefit from concave profiles to promote tissue thickening, while thick biotypes may tolerate convex designs without compromising soft tissues stability. Indeed, recent evidence suggests that the thin gingival phenotype is one of the main risk factors leading to marginal bone loss [18]. From this perspective, it appears to be of utmost importance to correctly manage the emergence profile of the implant-prosthetic restoration, especially in cases characterized by a thin phenotype.

Advances in digital workflows and CAD/CAM technology have revolutionized the customization of emergence profiles, enabling precise tailoring to the patient’s specific anatomical and functional needs [9]. These technological advancements contributed significantly to peri-implant health by reducing biological complications and enhancing the predictability of clinical outcomes [19,20]. Careful planning and the execution of emergence profile design are essential for achieving long-term success in implant-supported restorations.

## 3. Assessing Marginal Bone Level

Marginal bone level is a fundamental parameter in implant dentistry, as it reflects the biological and mechanical stability of dental implants and serves as a critical indicator of long-term success [21]. The preservation of marginal bone level is essential for maintaining implant function, supporting peri-implant soft tissues, and achieving predictable aesthetic outcomes. Stable peri-implant marginal bone is crucial for preventing complications such as implant thread exposure, increased bacterial colonization, peri-implantitis, and eventually implant failure. Numerous factors are capable of influencing marginal bone level, including implant design, surface characteristics, surgical technique, loading protocols, the emergence profile of the prosthesis, and the patient’s oral hygiene and systemic health [22,23,24]. The proper understanding and management of these factors play a pivotal role in minimizing bone loss, which is often associated with mechanical overload, inflammatory responses, or inadequate soft tissue adaptation.

The assessment of marginal bone level is typically performed using radiographic techniques, which are indispensable tools for monitoring peri-implant bone remodeling over time. Standardized periapical radiographs are commonly used for two-dimensional evaluations, whilst cone-beam computed tomography (CBCT) provides three-dimensional imaging for enhanced accuracy and spatial resolution [25]. Indeed, two-dimensional imaging allows one to evaluate the mesial and distal bone level but does not provide the assessment of the buccal and lingual bone plates. This limitation is particularly important in those cases where the goal is to evaluate the effects of regenerative procedures, such as bone augmentation performed in conjunction with delayed or immediate implant placement [26]. Radiographs are commonly taken immediately after implant placement to verify implant position and to establish a baseline to detect potential changes during follow-up visits. Quantitative assessment involves measuring the vertical distance from the implant platform or shoulder to the first visible bone-to-implant contact point. According to widely accepted criteria, bone loss within 1.5–2.0 mm in the first year after implant placement and no more than 0.2 mm annually thereafter is considered acceptable [27,28,29]. Significant deviations from these values may indicate complications such as peri-implantitis or excessive mechanical stress, requiring timely intervention.

Advances in digital imaging and software have enhanced the accuracy of bone-level measurements, allowing clinicians to detect subtle changes that may not be evident during clinical examination. Additionally, the longitudinal monitoring of marginal bone-level aids in the early identification of risk factors, such as insufficient soft tissue sealing or improper prosthetic design, enabling proactive adjustments to treatment plans. Overall, the meticulous evaluation and preservation of marginal bone level are indispensable for ensuring the long-term success and functionality of implant-supported restorations, as well as maintaining patient satisfaction and confidence in treatment outcomes.

## 4. Implications in Patient Treatments Outcomes

The implications of marginal bone-level stability on patient treatment outcomes in implant dentistry are far-reaching, directly impacting both the functional and aesthetic success of the restoration as well as the overall patient experience. Marginal bone stability is essential for maintaining the health of peri-implant tissues, which serve as a biological barrier protecting the implant from microbial invasion and inflammation. When marginal bone level is preserved, patients typically benefit from improved implant longevity, reduced risk of complications, and enhanced quality of life, as they experience fewer clinical issues and greater satisfaction with their treatment [22,30]. Conversely, bone loss beyond clinically acceptable thresholds—commonly defined as 1.5–2.0 mm in the first year and no more than 0.2 mm annually—can lead to several adverse outcomes, including peri-implantitis and aesthetic failures. This is particularly problematic in the anterior aesthetic region, where even minor alterations in soft and hard tissue contours can compromise the natural appearance of the restoration [29,31].

Marginal bone loss can also exacerbate systemic health conditions, as it is often linked to increased inflammatory burden. For patients with systemic conditions such as diabetes, osteoporosis, or a history of periodontitis, this can further complicate treatment and increase the risk of implant failure [32,33]. The interplay between marginal bone loss and systemic health underscores the need for personalized treatment strategies that consider the patient’s overall medical history, bone quality, and compliance with maintenance protocols. Effective management includes precise surgical and prosthetic planning, such as selecting implants with favorable designs (e.g., platform-switching configurations) and ensuring optimal emergence profiles to minimize stress on peri-implant tissues [4,9].

Advanced diagnostic technologies, including digital imaging and software tools, have significantly improved the ability to monitor marginal bone level over time. These tools allow for the accurate assessment of peri-implant bone changes, facilitating early intervention when necessary and reducing the likelihood of severe complications. Regular follow-up visits, thorough patient education on oral hygiene practices, and professional maintenance protocols are crucial in mitigating bone loss and enhancing long-term outcomes [34]. In sum, the preservation of the peri-implant marginal bone level is a cornerstone of implant dentistry, integral to achieving durable, functional, and aesthetically pleasing results while ensuring a positive impact on the patient’s overall health and satisfaction.

## 5. Implications for Clinicians

For clinicians, the implications of marginal bone level in implant dentistry are extensive and directly impact every stage of the treatment process, from initial assessment and planning to long-term maintenance and follow-up care. Marginal bone stability influences not only the longevity of the implant but also the surrounding soft tissues, the aesthetic outcomes, and the overall patient satisfaction. Therefore, clinicians must approach treatment with a comprehensive understanding of the factors affecting marginal bone level, including surgical techniques; prosthetic designs; loading protocols; and patient-specific variables such as bone quality, soft tissue biotype, systemic health, and oral hygiene practices [33,35].

During the planning phase, the clinician’s choice of implant system and design plays a critical role in bone preservation. Implants with platform-switching designs or surface modifications, such as roughened surfaces, have been shown to mitigate crestal bone loss by promoting better stress distribution and facilitating early osseointegration [4,32]. Advances in digital technology, including three-dimensional imaging and computer-aided surgical guides, allow for highly accurate implant placement and the customization of emergence profiles, reducing the risk of bone loss caused by biomechanical or biological factors. Surgical techniques that minimize trauma, such as flapless approaches, are particularly important in preserving marginal bone following implant placement.

**Figure 2:**
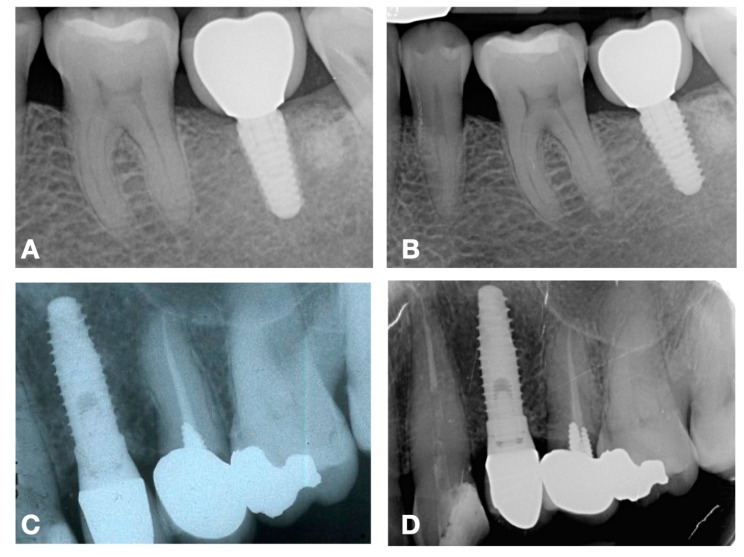
Radiographic evaluation of two different implant-prosthetic cases at baseline (**A**,**C**) and at 4 (**B**) and 8 (**D**) years of follow-up.

In the restorative phase, the timing and protocol of prosthetic loading are critical considerations. Excessive occlusal forces or premature loading can result in mechanical overload and inflammatory responses, leading to increased bone remodeling and subsequent peri-implant bone loss. Clinicians must carefully evaluate the patient’s occlusion and select prosthetic designs that distribute forces evenly to prevent excessive stress on peri-implant bone [36,37]. Long-term maintenance is equally important, as monitoring marginal bone level through radiographs and clinical assessments allows for early detection of potential issues, such as peri-implant peri-implantitis. Radiographic evaluation should be conducted at baseline and during regular follow-up visits (Figure 2), with precise measurement techniques to identify any deviations from expected patterns of bone remodeling [38].

Clinicians also bear the responsibility of educating patients on the importance of peri-implant hygiene and compliance with maintenance protocols. This involves clear communication about the role of daily oral hygiene, professional cleaning, and adherence to follow-up appointments in preventing complications that may compromise marginal bone stability. For patients with systemic conditions, additional precautions and tailored maintenance plans are essential to mitigate the heightened risk of bone loss [32]. The ability to preserve marginal bone level ultimately reflects the clinician’s expertise in integrating advanced technologies, evidence-based protocols, and patient-centered care strategies. The effective evaluation and management of the marginal bone level not only enhances the predictability of treatment outcomes but also reinforces patient trust and satisfaction, which are vital to the success of implant therapy in clinical practice.

## 6. Evidence from the Literature

Evidence from the literature is varied on the influence of prosthetic design, vertical platform discrepancies, and emergence angles (EAs) on the preservation of marginal bone level and peri-implant tissue health.

Yi et al. [39] found a significant correlation between prosthetic features and the development of peri-implantitis. According to their results, over-contoured restorations and EAs ≥ 30° were associated with higher marginal bone loss and the risk of developing peri-implantitis. Convex profiles and splinted-middle implants showed the highest prevalence, likely due to reduced hygiene accessibility. Tissue-level implants had lower peri-implantitis rates than bone-level implants, while the crown-to-implant (C/I) ratio was not a significant factor, potentially due to variations in implant length and depth. The authors concluded that avoiding over-contouring, optimizing EAs, and designing restorations might improve cleanability for better implant outcomes. Inoue et al. [40] provided a comprehensive multivariate analysis, revealing that taper joint connections and EAs between 20° and 40° were significantly associated with reduced marginal bone loss. The authors highlighted the biomechanical advantages of taper joints, which promote better load distribution and minimize micromovement at the implant–abutment interface, reducing the risk of bone resorption. Additionally, 20°–40° EAs allowed for optimal soft tissue adaptation and stress distribution, further contributing to marginal bone stability. Interestingly, factors such as the retention type (cemented vs. screw-retained) and abutment material showed less consistent effects on bone loss, indicating a need for individualized assessment in clinical decision-making. Other articles focused on this kind of connection, showing favorable mechanical results from in vitro and in vivo models [41,42,43,44].

Lin et al. [45] explored the morphology of peri-implant tissues around implant-prosthetic restorations with varying EAs, particularly in cases where free gingival grafting was performed to augment peri-implant soft tissues. The study found that EAs exceeding 30° were associated with increased marginal bone loss and reduced vertical soft tissue thickness, further compromising the peri-implant seal. These results suggested the importance of designing conservative emergence profiles to facilitate effective soft tissue integration and prevent plaque accumulation, both of which are critical for maintaining crestal bone stability.

The findings by Montaruli et al. [46] were also in agreement, reporting a critical role of prosthetic factors in minimizing marginal bone loss around implants. While internal connections showed trends for improved short-term stability, external connections remain widely used for their established longevity. Cement-retained restorations with EAs over 30° were strongly linked to increased bone loss, having a greater impact than platform switching. Implant length also influenced outcomes, with shorter implants showing reduced marginal bone remodeling, while factors like crown-to-implant ratio and healing protocol showed no significant correlation. Another study by Lin et al. [47] focused on the effects of vertical platform discrepancies and splinting on adjacent implants, finding that a platform height difference exceeding 0.5 mm was significantly associated with higher marginal bone loss. Furthermore, splinted crowns demonstrated greater bone loss compared to non-splinted restorations, suggesting that splinting can exacerbate stress transfer and hinder effective hygiene access. This study emphasized the importance of achieving precise implant positioning and minimizing discrepancies during placement to optimize long-term outcomes. The findings also highlighted that restorations such as three splinted adjacent crowns were associated with the highest rates of bone loss, reinforcing the need for the careful consideration of prosthetic design in multi-implant cases.

As a matter of fact, the exact causes of implant-threatening marginal bone loss are unclear, with other factors potentially contributing to this effect, including implant design and surface [48], as well as emergency angle and profile.

Interestingly, Katafuchi et al. [49] reported that for bone-level implants, a restoration with EAs exceeding 30° on at least one proximal surface was associated with a higher occurrence of peri-implantitis, particularly when combined with a convex profile. Restorations with wide EAs and convex profiles were reported to negatively affect peri-implant health and heighten the likelihood of developing peri-implantitis. To reduce this risk, shallower EAs paired with a straight or concave profile were thus recommended. On the other hand, tissue-level implants showed no correlation between EAs or profiles and the incidence of peri-implantitis, meaning no specific recommendations can be made regarding these factors for tissue-level implants.

However, other studies report an absence of a relationship between MBL and prosthetic EAs.

Hentenaar et al. [50] investigated the impact of cervical crown contour on MBL and peri-implant soft tissue health around platform-switched, posteriorly placed, two-piece implants over a 5-year period. Mean crown angles did not exceed 18.7°, and no correlation was observed between EAs and marginal bone loss or peri-implantitis. Clinical parameters, including gingival health and probing depths, indicated high levels of peri-implant health, with no cases of peri-implantitis reported. The authors concluded that platform-switched implants, when restored with customized superstructures, maintained favorable hard and soft tissue conditions regardless of crown contour.

Lops et al. [51] observed bone stability with mean EAs of 45°, differing from findings that suggested lower angles (20°–40°) might be more favorable. As no significant differences in bleeding and plaque indices were found, the authors suggested that EAs > 30° might not pose a risk for peri-implant health. Contrasting results, like those from Katafuchi et al. [49], linked higher EAs with increased risk to develop peri-implantitis, particularly in non-platform-switched implants. However, in this study, marginal bone loss was minimal (0.06 mm) regardless of EAs, aligning with previous findings on implants with platform-switching designs. The study’s limitations included the inability to assess the vestibular aspect of the bone and control confounding factors affecting bone stability. Despite these constraints, the findings suggested that, with stable implant–abutment connections, EAs between 30° and 50° did not significantly affect MBL stability.

Differences in the marginal bone loss were not detected by Wang and collaborators [52]. Their study examined the impact of emergence profile design, guided by the mucosal width-to-height (W/H) ratio, on soft tissue stability and bone resorption around molar implants. Using a baseline supracrestal soft tissue thickness of ≥2 mm, the experimental group with a modified emergence profile (W/H ratio of 1.3) exhibited significantly less gingival recession and reduced probing depth (PD) compared to the control group. Smaller EAs (32.4°), measured with precise digital modeling, further supported gingival margin stability, consistent with findings linking excessive angles to peri-implantitis. Although bone loss differences were not statistically significant, reduced PD minimized biological risks. Variations in W/H ratios were attributed to differences in implant-system connections, with higher stability yielding better outcomes.

Similarly, Kou et al. [53] did not find a correlation between EAs > 30° with peri-implantitis and early MBL, although both bone-level and tissue-level implants were included in the study. However, site-specific variations were noted, with 80% of anterior implants having angles below 30°, compared to 60% of molar implants exceeding this threshold.

Finally, a study by Cinquini et al. [54] found no significant influence of the prosthetic EAs on marginal bone loss over time, regardless of whether alveolar ridge preservation procedures were performed or the post-extraction site underwent spontaneous healing.

The results of this review should be interpreted with caution. Indeed, the main limitation of this review is its narrative design, as no systematic approach was adopted, and no meta-analyses were conducted. However, although the current literature on the influence of prosthetic emergence profile remains controversial, it is essential to prioritize cleanability and minimize plaque accumulation for successful implant-prosthetic restoration. Further research should focus on improving the choice of emergence profile, optimizing biomechanical stability, ensuring even force distribution, and preventing stress concentration on surrounding tissues. The EAs should promote soft tissue adaptation, maintaining a stable mucosal seal while minimizing plaque accumulation to facilitate hygiene and long-term maintenance. In the anterior region, aesthetic integration is needed to achieve a natural transition between the restoration and soft tissues. Monitoring both marginal bone level and soft tissue response over time may also help the assessment of peri-implant health and stability. Long-term follow-up studies are needed to assess how different profiles might influence tissue stability and implant success rates over time.

## 7. Conclusions

The relationship between EAs and marginal bone loss is still debated in the literature, as many factors appear to concur to maintain peri-implant tissues stability. Indeed, the performance of correct oral hygiene procedures, as well as a close follow-up, may promote the achievement of stable and optimal long-term results. The early detection of variations in peri-implant health status, along with the identification of mechanical or prosthetic failures, may enable timely intervention to prevent further complications.

## Data Availability

Not applicable.
